# Identification and characterisation of Dof transcription factors in the cucumber genome

**DOI:** 10.1038/srep23072

**Published:** 2016-03-16

**Authors:** Chang-long Wen, Qing Cheng, Liqun Zhao, Aijun Mao, Jingjing Yang, Shuancang Yu, Yiqun Weng, Yong Xu

**Affiliations:** 1 Beijing Vegetable Research Center (BVRC), Beijing Academy of Agricultural and Forestry Sciences, National Engineering Research Center for Vegetables, Beijing 100097, China; 2 Beijing Key Laboratory of Vegetable Germplasms Improvement, Beijing 100097, China; 3 Beijing Agricultural extension station, Beijing 100029, China; 4 USDA-ARS, Vegetable Crops Research Unit, Horticulture Department, University of Wisconsin, Madison, WI 53706, USA

## Abstract

Cucumber is vulnerable to many foliage diseases. Recent studies reported cloning of candidate genes for several diseases in cucumber; however, the exact defence mechanisms remain unclear. Dof genes have been shown to play significant roles in plant growth, development, and responses to biotic and abiotic stresses. *Dof* genes coding for plant-specific transcription factors can promote large-scale expression of defence-related genes at whole genome level. The genes in the family have been identified and characterized in several plant species, but not in cucumber. In the present study, we identified 36 *CsDof* members from the cucumber draft genomes which could be classified into eight groups. The proportions of the *CsDof* family genes, duplication events, chromosomal locations, cis-elements and miRNA target sites were comprehensively investigated. Consequently, we analysed the expression patterns of *CsDof* genes in specific tissues and their response to two biotic stresses (watermelon mosaic virus and downy mildew). These results indicated that *CsDof* may be involved in resistance to biotic stresses in cucumber.

Cucumber is a major vegetable crop worldwide^1^,^2^,^3^, which belongs to the *Cucurbitaceae* family and is vulnerable to infection or infestation by many bacterial, fungal and viral pathogens or insects such as *Fusarium oxysporum* (Fusarium wilt, FW)^4^,^5^, *Podosphaera fusca* (powdery mildew, PM)^6^, *Pseudoperonospora cubensis* (downy mildew, DM)^7^, Zucchini yellow mosaic virus (ZYMV)^8^, *Corynespora cassiicola* (target leaf spot, TLS)^9^, and *Cladosporium cucumerinum* (scab)^10^,^11^. These biotic stresses pose a substantial threat to yield and food security. In recent years, several resistance genes or resistance gene candidates have been cloned in cucumbers; however, the defence mechanisms remain unknown. Cucumber was the first vegetable crop with a draft genome sequence^1^,^2^. The publicly available whole-genome and many transcriptome sequences of cucumber provide a good opportunity to study disease resistance mechanisms, especially from a whole-genome perspective.

The Dof (DNA-binding with one finger) proteins are a group of plant-specific transcription factors (TFs) that have typically 200–400 amino acids with a highly conserved 52 amino acid Dof DNA-binding domain at the N-terminal. The Dof domain has a Cys2/Cys2 Zn^2+^ finger structure that recognises the specific cis-element of (AT)/AAAG in the promoter region of their target genes^12^,^13^,^14^. The C-terminal of the Dof proteins contains a transcriptional regulation domain with diverse functions including interaction with various regulatory proteins and activation of gene expression^15^. The N- and C- terminal regions of Dof protein may interact with various regulatory proteins or intercept signals to mediate the activation or repression of the target genes^16^.

The Dof TFs have been shown to play important roles in many biological processes, such as synthesis of seed storage protein, seed development and germination, photosynthetic processes and flowering^17^. Dof TFs participate in various physiological processes such as the cell cycle, adaxial-abaxial polarity, plant hormonal signalling, phytochrome and cryptochrome signalling, nitrogen use, biotic and abiotic stress tolerance^18^. The Dof members could play roles in the regulation of secondary metabolic processes, such as the biosynthesis of glucosinolates and flavonoids^19^,^20^, and cell cycle regulation^21^. In *Arabidopsis*, the *Dof* genes *DAG1* and *DAG2* are associated with seed germination^22^,^23^, and *CDF1*, *CDF2*, and *CDF3* control photoperiodic flowering^24^. Some other *Dof* genes (for example, *AtDof2.4, AtDof5.8* and *AtDof5.6*/*HCA2*) are specifically expressed in the early stage of vascular tissue development^25^,^26^. In tomato, *StDof1* is specifically expressed in epidermal fragments of guard cells; *StDof1* can interact with the promoter of the *KST1* gene^13^. Rice *OsDof3* was reported to be involved in gibberellins-regulated expression^27^. Maize *Dof1* and *Dof2* were confirmed to be activators of genes associated with carbohydrate metabolism and contained the C_4_ key photosynthetic gene phosphoenolpyruvate carboxylase (PEPC)^28^.

Dof transcription factors have been functionally characterised in Arabidopsis^22^,^23^ and a number of crops such as tomato^18^, rice^27^ and soybean^29^. However, no such information is available in cucumber. The objective of the present study was to conduct a genome-wide characterisation of *Dof* gene families in the cucumber genome. We identified 36 CsDof members in the cucumber draft genome, which were classified into eight subgroups. The proportions of the *CsDof* family genes, duplication events, and chromosomal locations were investigated. We also analysed the expression patterns of *CsDof* members in various tissues and the response to inoculation of the watermelon mosaic virus (WMV) as well as downy mildew (DM) pathogens. Our study provided novel insights into the stress responses of *CsDof* genes to these two important cucumber pathogens and deepens our understanding of the structure and function of *CsDof* genes in the cucumber genome.

## Results

### Identification of *CsDof* homologues in the cucumber genome

To identify the Dof transcription factor coding genes in the cucumber genome, we used the HHM profile of the Dof domain (PF02701) as a query to perform an HMMER search (http://hmmer.janelia.org/) against the 9930 and Gy14 cucumber draft genomes. Thirty-six *Dof* gene sequences were identified from the HMMER database (http://www.ebi.ac.uk/Tools/hmmer/search/hmmsearch). We then performed SMART search (http://smart.emblheidelberg.de/) to confirm the presence of the conserved Dof domain which confirmed the existence of 36 putative *CsDof* genes in the cucumber genome. For convenience, the 36 *CsDof* genes were assigned names from *CsDof01* to *CsDof36* based on their chromosomal locations (Table 1). These *CsDof* genes encoded predicted peptides ranging from 150 to 503 aa with the pI value from 4.8 to 9.6 and the molecular weight from 16.9 to 55.2 kD. All CsDof proteins were predicted to be localised in the nucleus except for CsDof30 which was extracellular (Table 1). In addition, using BLASTN, we found nine *CsDof* genes (*CsDof02, CsDof04, CsDof10, CsDof11, CsDof13, CsDof16, CsDof18, CsDof28 and CsDof36*) showed polymorphisms between 9930 and Gy14. The SNPs and their locations within each gene are presented in Supplementary Table S1.

### Phylogenetic analysis

**Figure 1:**
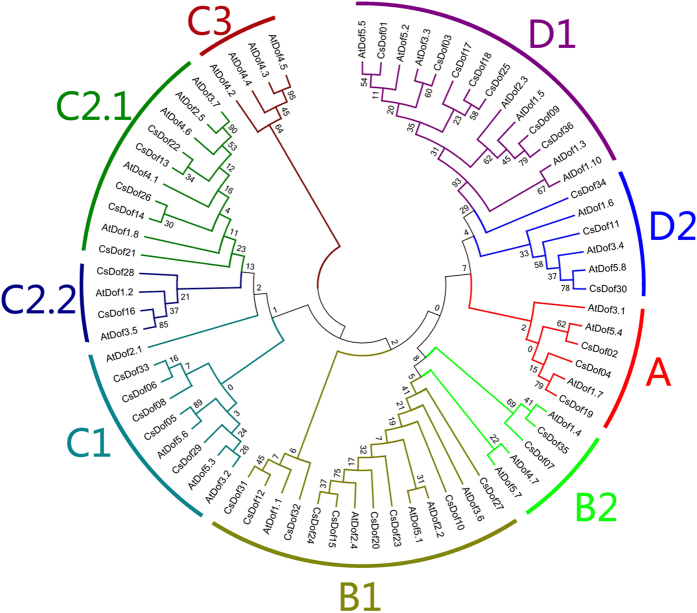
Phylogenetic tree of the cucumber and Arabidopsis Dof gene families. The unrooted phylogenetic tree was constructed using MEGA 6.0 and the Neighbour-Joining method. The bootstrap test was performed with 1000 iterations. The nine subclades were shown in different colours.

To clarify the phylogenetic relationships among 36 *CsDof* genes, a phylogenetic tree was constructed based on the alignment of the CsDof domain sequences (Supplementary Fig. S1). The neighbour joining (NJ) phylogenetic tree divided the *CsDof* homologs into four groups (A to D), which could be further classified into 8 subgroups: A, B1, B2, C1, C2.1, C2.2, D1, and D2. The bootstrap values are shown in Fig. 1. Subgroup B1 contained the most *CsDof* family members (9 genes, or 25%); B2 and C2.2 constituted the smallest clade with only 2 members each. Groups C2.1 and C2.2 contained 5 *CsDof* genes (13.9%); group D1 showed 7 members representing 19.4% of the total *Dof* genes. All 36 *CsDof* genes showed the conserved Cys2/Cys2 Zn^2+^ finger in the Dof domain (Supplementary Fig. S1).

### Chromosomal locations and duplications of Dof members

**Figure 2:**
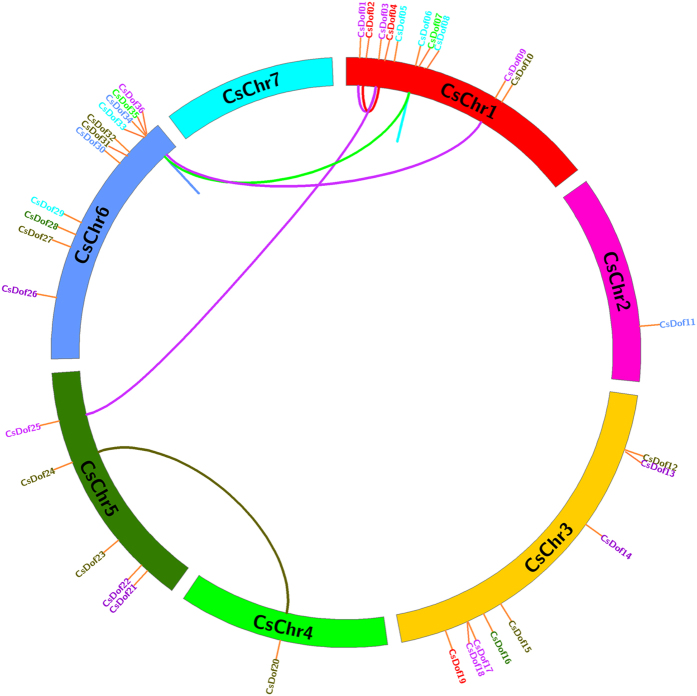
Distribution of 36 *CsDof* genes onto seven cucumber chromosomes. Graphical representation of physical locations for each *CsDof* gene on cucumber chromosomes. The *CsDof* duplications genes were connected with a line. The *CsDof06*/*CsDof07* and *CsDof34*/*CsDof35* were tandem duplicated genes, and the *CsDof01*/*CsDof03, CsDof02*/*CsDof04, CsDof03*/*CsDof25, CsDof07*/*CsDof35, CsDof09*/*CsDof36* and *CsDof20*/*CsDof24* were undergoing segmental duplication events.

As shown in Fig. 2, the 36 *CsDof* genes were dispersed on 6 of the 7 cucumber chromosomes (none in chromosome 7). Chromosome 6 harboured the most (11 of 36) *Dof* genes, whereas 10, 8, 5, 1 and 1 were mapped on chromosomes 1, 3, 5, 2 and 4, respectively (Fig. 2).

In this study, the *CsDof* genes duplications were analysed using the CoGe website. Two pairs of tandem duplicated genes were identified (*CsDof06*/*CsDof07* and *CsDof34*/*CsDof35)*, and 6 pairs of segmental duplication genes were observed (*CsDof01*/*CsDof03, CsDof02*/*CsDof04, CsDof03*/*CsDof25, CsDof07*/*CsDof35, CsDof09*/*CsDof36* and *CsDof20*/*CsDof24*) (Fig. 2). To trace the dates of the duplication blocks, we estimated the Ks/Ka distances and ratios. The segmental duplications of the *CsDof* genes in cucumber originated from approximately 4.639 Mya (million years ago, Ks = 0.6031) to 11.66 Mya (Ks = 1.5161) with an average of 9.079 Mya (Ks = 1.180). The Ks of the tandem duplication of *CsDof06* and *CsDof07* was 1.8735, which dated the duplication event at 14.41 Mya; that the tandem duplication of *CsDof34* and *CsDof35* was 1.3398, dating the duplication event at 10.31 Mya (Supplementary Table S2).

### Gene structure and conserved motifs analysis

**Figure 3:**
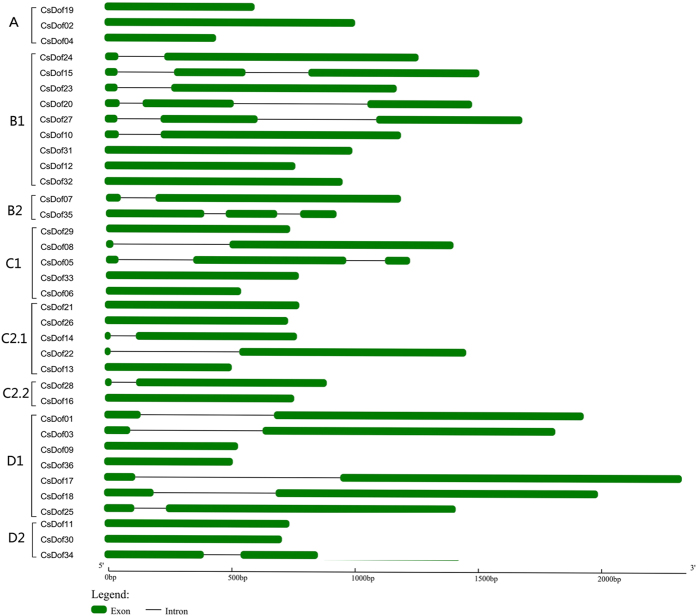
Exon-intron structures of CsDof genes in cucumber genome. All of the *CsDof* genes were divided into four groups or eight subgroups. The green bars indicated the exons, and the black lines indicated the introns.

In order to investigate the characterization of exon-intron structure, all of the *CsDof* genes were analyzed by using the Gene Structure Display Server. As shown in Fig. 3, the predicted number of exons among the 36 *CsDof* genes were relatively fewer, and they varied from one to three with 17 members having one and 14 with two. Five (*CsDof 05, CsDof15*, *CsDof 20*, *CsDof 27*, and *CsDof 35*) had three exons. Furthermore, some *CsDof* genes within the same subgroup demonstrated similar exon/intron structure patterns in terms of exon number. For instance, all *CsDof* genes in subgroup A had no introns. The two exons *CsDof* gene were mainly in group C (four in C1, C2.1 and C2.2) and D (five in D1 and one in D2). The majority three excons were belonging in group B (B1 and B2). These similar structure feature may be related to their functions in cucumber genome (Fig. 3).

To obtain insights into the diversity of motif compositions in CsDof proteins, these proteins were assessed using the MEME programme. A total of 15 conserved motifs were identified (Supplementary Table S3; Fig. S2). These motifs are represented in their relative location within the protein. Motif 1 was uniformly observed in all Dof proteins and was confirmed to be the conserved Dof domain. Moreover, the CsDofs in each subgroup had several special motifs at C-terminal regions, suggesting that they had a similar function in the CsDof members within the same subgroups (Supplementary Fig. S2). Groups A, C2.2 and D2 showed one conserved motif (motif 1). Group D1 members contained motif 4 and motif 12, and Group B1 contained motif 2 and motif 7.

### Cis-element analysis of *CsDof* genes

In the *CsDof* genes promoter region, many key defence cis-elements (such as ACGTABOX, ASF1MOTIFCAMV, and WBOXATNPR1) were identified. Other key elements included those in hormone signalling, such as ARFAT (auxin), ASF1MOTIFCAMVT (auxin), ERELEE4 (ethylene), GAREAT (gibberellin), LECPLEACS2 (ethylene), MYBGAHV (gibberellin), NTBBF1ARROLB (auxin), and TATCCAOSAMY (gibberellin). Some *CsDof* genes harboured additional transcription factor cis-regulatory elements, e.g., MYB1AT, MYB1AT, MYB1AT, MYBGAHV, MYBCOREATCYCB1, and WRKY71OS (Supplementary Table S4). All *CsDof* gene promoters contained the conserved elements DOFCOREZM, suggesting that these *CsDof* genes could be regulated by themselves. Additionally, an *in silico* analysis showed that the *CsDof* gene promoters maintained several tissue-specific elements. Some examples included root-specific (ROOTMOTIFTAPOX1, RAV1AAT and SP8BFIBSP8BIB), leaf-specific (CCA1ATLHCB1, DOFCOREZM, GATABOX, GT1CONSENSUS, IBOXCORE and RAV1AAT) and flower-specific (CARGCW8GAT) responsive elements (Supplementary Table S4).

### Regulated miRNAs

Among the 36 *CsDof* genes, 15 were targeted by 20 miRNAs. *CsDof21* had the most abundant transcript, which was targeted by 6 plant miRNAs (miR5021, miR5658, miR2673a, miR2673b, miR2673, and miR7494b) (Supplementary Table S5). A sequence analysis of *CsDof26* implied that the 188–288 bp region may be the target site of miR319a. The miR5658 targeted *CsDof05*, *CsDof08*, *CsDof20* and *CsDof21*. The *CsDof28* was targeted by 5 plant miRNAs (miR831, miR831-5p, miR393b-3p, miR393b-3p and miR3434-3p) (Supplementary Table S5).

### Tissue-specific expression of *CsDof* genes

**Figure 4:**
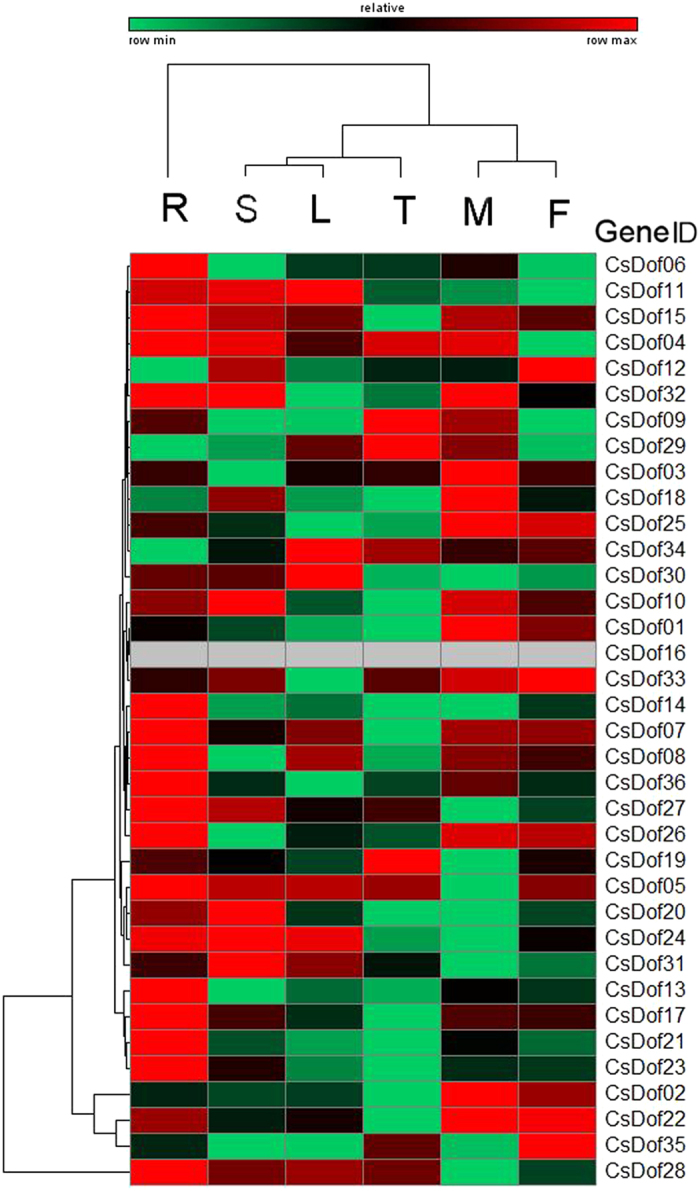
Tissue-specific digital expression profiles of *CsDof* transcription factors. The *CsDof* genes were listed on the right of the expression array, and the colour scale was shown at the top. The higher expression for each gene was presented in red; otherwise, green was used. The genes with an RPKM equal to 0 were not used in this array. The capital letters indicated the following: R = root; S = stem; L = leaf; T = tendril; M = male flower; F = female flower.

From analysis of publicly available transcriptome data, we observed 17 of 36 *CsDofs* that had a relatively high expression in root tissues including *CsDof04*, *CsDof05, CsDof06, CsDof07, CsDof08, CsDof11, CsDof13, CsDof14, CsDof15, CsDof17, CsDof21, CsDof23, CsDof24, CsDof26, CsDof27, CsDof28* and *CsDof32*. Among the 17, *CsDof* genes, *CsDof06*, *CsDof13, CsDof14, CsDof17, CsDof21* and *CsDof23* were only expressed in the root, suggesting that these six genes may play specific roles in root development. Moreover, *CsDof04, CsDof10, CsDof11, CsDof15, CsDof20, CsDof24, CsDof31* and *CsDof32* were specifically expressed in the stems (Fig. 4). The expressions of five genes, *CsDof01, CsDof02, CsDof03, CsDof18*, and *CsDof25*, were specific to male flowers, whereas *CsDof12, CsDof33* and *CsDof35* were specifically expressed in female flowers. Finally, 3 (*CsDof11*, *CsDof30* and *CsDof34*) and 3 (*CsDof09*, *CsDof19* and *CsDof29*) genes were specifically expressed in the leaves and tendrils, respectively (Fig. 4).

### Responses to DM and WMV pathogen inoculations

**Figure 5:**
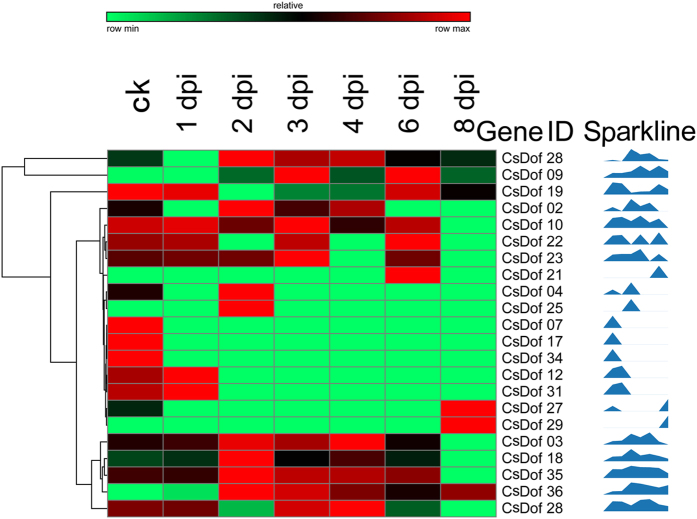
Expression profile of *CsDof* transcription factors under downy mildew. The expression colour scale was shown at the top. The 22 *CsDof* genes were observed in transcriptome data with DM inoculation, and the sparkline presented the expression variation. Higher expression for each gene was presented in red; otherwise, green was used. The genes with an RPKM equal to 0 were not used in this array. The columns indicated the changes in the *CsDof* gene expression levels at various time points (1, 2, 3, 4, 6 and 8 dpi; Ck was treated with MOCK) under DM stress.

Of 36 *CsDof* genes, 22 were responsive to DM pathogen inoculation (Fig. 5). Of these, four *CsDof* genes (*CsDof07*, *CsDof17*, *CsDof19* and *CsDof34*) had lower expression as compared with the control, whereas two genes (*CsDof27* and *CsDof29*) exhibited higher expression than in the control after inoculation. Additionally, five members (*CsDof03*, *CsDof18*, *CsDof28*, *CsDof35* and *CsDof36*) were first up- and then down- regulated. Moreover, four genes (*CsDof10*, *CsDof12*, *CsDof19* and *CsDof31*) were the highest in expression on the first day after DM inoculation, whereas the expression of seven genes (*CsDof02*, *CsDof04*, *CsDof18*, *CsDof25*, *CsDof28*, *CsDof35* and *CsDof36*) peaked on the second day after inoculation.

**Figure 6:**
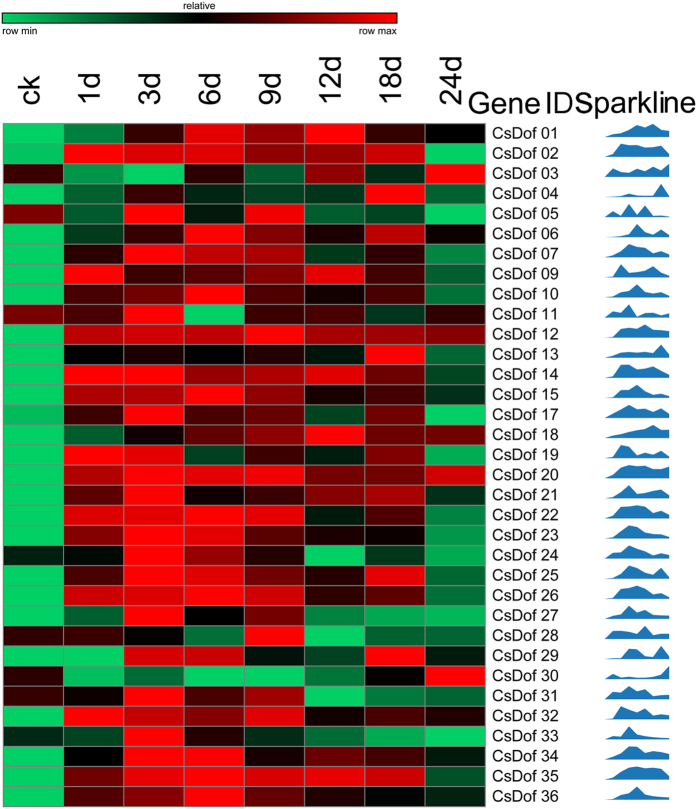
Expression profiling of *CsDof* genes under WMV stress. The 34 *CsDof* genes were determined to have varied expression after WMV inoculation via qRT-PCR, and the sparkline presents the expression variation. The expression colour scale was shown at the top. A higher expression for each gene was presented in red; otherwise, green was used. The columns indicate the changes in *CsDof* gene expression levels at various time points (1 d, 3 d, 6 d, 9 d, 12 d, 18 d and 24 d under WMV inoculation; Ck was treated with MOCK).

Quantitative real-time PCR was conducted for these 36 *CsDof* genes to study their expression upon inoculation of the WMV viral pathogen (Fig. 6). Among them, 34 were up- or down-regulated after WMV inoculation, and no changes were observed for *CsDof08* or *CsDof16*. Three genes (*CsDof02, CsDof14* and *CsDof35*) were initially up- regulated but down- regulated after 24 d. The expressions of ten genes (*CsDof02, CsDof09, CsDof12, CsDof14, CsDof15, CsDof19, CsDof20, CsDof22, CsDof26* and *CsDof32*) increased on the first day after inoculation. Overall, 20 *CsDof* genes exhibited higher expression on the third day after WMV inoculation. *CsDof28, CsDof30* and *CsDof33* were highly expressed at 3 d, 9 d and 24 d after inoculation, respectively, whereas the expression of *CsDof04* and *CsDof13* peaked at 18 d after inoculation.

## Discussion

In recent years, gene family analysis has become an important approach to understand gene structure, function, and evolution. The *Dof* genes are plant-specific transcription factors that are involved in various biological processes and are ubiquitous in many plant species. The function and evolution of *Dof* genes have been thoroughly studied in *Arabidopsis* (36 *AtDof* genes), rice (30 *OsDof* genes)^30^, soybean^29^ (78 *GmDof* genes), Chinese cabbage^15^ (76 *BraDof* genes), potato^17^ (35 *StDof* genes), pigeonpea^31^ (38 *CcDof* genes), and tomato^18^ (34 *SlDof* genes). Here, we conducted a comprehensive analysis of the CsDof family in cucumber to determine their potential functions in response to biotic stresses.

We identified 36 putative *CsDof* genes in the cucumber genome; the number of CsDof homologues was largely similar to that found in *Arabidopsis* or rice^30^. The 36 *CsDof* genes were classified into four groups (A to D) and eight subgroups (A, B1, B2, C1, C2.1, C2.2, D1, and D2) (Fig. 1). In *Arabidopsis*, all 36 *AtDof* genes were divided into four groups and nine subgroups (A, B1, B2, C1, C2.1, C2.2, C3, D1, and D2)^30^; the *CsDof* genes were not found in subgroup C3. In cucumber, subgroup B1 contained nine *CsDof* genes; only five were noted in *Arabidopsis*. Subgroup B2 had two *CsDof* members but three *AtDof* genes. These results indicated that *CsDof* and *AtDof* genes underwent different duplication events.

Similar to that found in rice and *Arabidopsis*^30^, the cucumber *Dof* genes had few introns (0–2) in each gene (Fig. 3). A motif analysis indicated that motif 1 was uniformly observed in all Dof proteins (Supplementary Fig. S2). Similar to the results from *Arabidopsis*, rice^30^ and tomato^18^, this phenomenon suggested that CsDof transcription factors were evolutionarily conserved in plants.

The whole-genome duplications, segmental/tandem duplication and transposition events were crucial for gene family expansion. Cucumber did not experience a recent whole-genome duplication^1^; thus, the remainder duplications would play crucial roles in gene expansion. In this study, we found 8 pairs of *CsDof* genes that had undergone duplications (Fig. 2). Intriguingly, we observed six pairs of segmental duplication and two pairs of tandem duplication events in *CsDof* genes. This indicated that *CsDof* gene segmental duplication is predominant in the evolution of cucumber and that tandem duplication is involved^18^. The duplication divergence of monocots and dicots occurred approximately 170–235 Mya^32^. This study showed that the mean date of *CsDof* genes tandem duplication events was at 12.36 Mya, whereas the mean date of segmental duplication events was at 9.079, which showed that the segmental/tandem duplication occurred after the divergence of the monocot-dicot split and that the tandem duplication occurred prior to the segmental duplication in cucumber *CsDof* genes.

Gene expression patterns provided important clues for gene function; thus, we conducted a digital gene expression analysis for duplicated *CsDof* genes in the root, leaf, stem, tendril and flower tissues using public RNA-seq data^33^. The Ka/Ks ratio analysis indicated that the *CsDof* genes had divergent functions after the duplication events. All tested 8 pairs of genes showed distinct expression patterns both in a tissue-specific and biotic responsive manner. For example, in one pair of segmental duplication *CsDof* genes, *CsDof07* was highly expressed in the root and *CsDof35* was highly expressed in female flowers (Fig. 4). The expression of *CsDof07* was relatively low and reached maximum at the third day after WMV inoculation; however, *CsDof35* was expressed at a higher level and peaked at the sixth day after WMV inoculation (Fig. 6). The tandemly duplicated *CsDof* genes were very differently expressed. *CsDof34* and *CsDof35* had specific expressions in the leaves and female flowers, respectively, and *CsDof34* had the highest expression at day zero (Mock treatment) under DM inoculations; however, *CsDof35* reached its peak expression at 2 d after DM inoculation (Fig. 5). These results indicated that the duplicated *CsDof* genes may play crucial and diversifying roles in plant development.

Dof TFs have been shown to play crucial roles in the regulatory networks of plant defence, including responses to diverse biotic and abiotic stresses^15^,^34^,^35^. In tobacco, the *Sar8.2b* gene can be promoted by the Dof transcription factor, which is involved in systemic acquired resistance^36^. In tomato, the Dof transcription factor was found to regulate fungus resistance through the *ACBP3* gene, which promoted autophagy-mediated leaf senescence and conferred resistance to *Pseudomonas syringae pv*. tomato DC3000^37^. In barley seeds, the Dof transcription factors play a role in biotic stress tolerance based on their association with the cystatin gene^38^. The *Dof* gene may play an indirect role in responding to biotic stresses, and we speculated that the CsDof transcript factor is involved in resistance through a direct or indirect manner; these may thus promote different target resistant genes in cucumber. In this study, we investigated the response patterns of *CsDof* genes to DM and WMV inoculations. We found that 19 and 34 *Dof* genes were up regulated after DM and WMV inoculations, respectively, suggesting that most of these *Dof* genes may play positive roles in host defence responses; however, additional work is needed to confirm their roles.

To summarise, in this study, we reported a comprehensive analysis of CsDof transcription factor genes in the cucumber genome. The 36 CsDof genes were categorised into eight subgroups, and the structural and functional properties of each CsDof member were characterised. Most of the *CsDof* genes were induced by biotic stresses. Our work will assist in understanding the roles of these *CsDof* transcription factors in response to biotic stresses and their potential interactions with defence-related genes in the disease resistance network.

## Methods

### Genome-wide identification of *CsDof* homologue sequences

The conserved Dof domain based on a Hidden Markov Model (HMM) (PF02701) was downloaded from the Pfam protein family database (http://pfam.sanger.ac.uk/). To identify the Dof transcription factor coding genes of *Cucumis sativus*, we used the HHM profile of the Dof domain as a query to perform a HMMER search (http://hmmer.janelia.org/) against the cucumber genome databases (http://www.icugi.org/; http://cucumber.genomics.org.cn/; http://wenglab.horticulture.wisc.edu/). All non-redundant sequences encoding complete Dof domains were considered to be putative *Dof* genes. Each non-redundant sequence was double checked for the presence of the conserved Dof domain using a SMART search (http://smart.emblheidelberg.de/). The candidate cucumber *CsDof* genes were named based on their distribution on the seven cucumber chromosomes. The ExPASy server (http://web.expasy.org/compute_pi/)^39^ was used to compute the pI and molecular weight of the identified CsDof proteins. We performed a nuclear localisation signal (NLS) analysis prediction of the Dof protein on the website (http://cello.life.nctu.edu.tw/).

### Phylogenetic characterisation of *CsDof* homologs

For a phylogenetic analysis of the plant *Dof* gene family, nucleotide or protein sequences from *Arabidopsis* were obtained from previous studies^30^. The information of Dof genes in *Arabidopsis* are presented in Table S6. Multiple sequence alignments were conducted on the amino acid sequences of Dof proteins from cucumber and *Arabidopsis* using ClusterW with default settings. Subsequently, MEGA 6.0 software was employed to construct an unrooted phylogenetic tree based on alignments using the Neighbour-Joining (NJ) method with the following parameters: JTTmodel, pairwise gap deletion and 1000 bootstraps^40^. Furthermore, maximum likelihood, minimal evolution and PhyML methods were also applied for the tree construction to validate the results of the NJ method.

### Chromosomal locations and duplications

Chromosomal locations for each *CsDof* gene were determined via BLASTP search against the cucumber genome databases with default settings. Tandem duplications and segmental duplications in the cucumber genome were analysed using the website tool CoGe (https://genomevolution.org/CoGe/)^41^, and the duplicated genes were linked with coloured lines which created by using the Circos software^42^ (http://circos.ca/).

To estimate the synonymous and non-synonymous substitution rates, we used the software DnaSp^43^. The time (million years ago, Mya) of duplication and divergence of each *CsDof* genes were estimated using a synonymous mutation rate of λ substitutions per synonymous site per year, T = Ks/2λ (λ = 6.5610 e-9)^33^.

### Gene structure analysis and conserved motif identification

The exon-intron organisations of the genes were determined using the Gene Structure Display Server (http://gsds.cbi.pku.edu.cn) through a comparison of their full-length cDNA or predicted coding sequence (CDS)^44^. The motifs of the Dof protein sequences were statistically identified using the MEME programme (http://meme-suite.org/tools/meme) with the motif length set to 6–100 and motif sites to 2–120. The maximum number of motifs was set to 15, the distribution of one single motif was “any number of repetitions” and the other parameter was “search given strand only”.

### Cis-element and miRNA target analysis

The mature miRNAs sequences were downloaded from miRBase v20.0 (http://www.mirbase.org) and PMRD (http://bioinformatics.cau.edu.cn/PMRD). Known cucumber miRNAs were used to identify the miRNAs target genes in the *CsDof* families. The prediction was conducted using the Plant Small RNA Target Analysis Server (psRNA Target: http://plantgrn.noble.org/psRNATarget) with default parameters. Alignment between all known plant miRNA and their potential *CsDofs* targets were evaluated using previously described parameters^45^. To identify the putative cis-acting regulatory elements presented in the promoter regions of *CsDof* genes, nucleotide sequences of 2000 bp upstream regions from the translational start codon (ATG) were retrieved from the PGSC database. An *in silico* promoter analysis was carried out using the PLACE database (http://www.dna.affrc.go.jp/PLACE/signalscan.html).

### Tissue-specific expression

The cucumber genome-wide digital gene expression was assessed as described by Baloglu *et al*.^33^. Illumina sequencing reads from RNA-Seq studies were retrieved from a public repository database (SRA, Sequence Read Archive) with the following accession numbers: SRR351499 (cucumber root tissue), SRR351905 (cucumber stem tissue), SRR351906 (cucumber leaf tissue), SRR351908 (cucumber male flower tissue), SRR351912 (cucumber female flower tissue) and SRR351910 (cucumber tendril tissue). All transcript data were analysed using Gene-E 3.0.240 (www.broadinstitute.org/cancer/software/GENE-E).

### Response to biotic stresses

To investigate the expression profiling of *CsDof* genes in response to downy mildew inoculation, the GEO data were downloaded from the NCBI PubMed database (http://www.ncbi.nlm.nih.gov/pubmed/) based on previous literature^46^. The data were used to draw heatmaps with Gene-E 3.0.240 (www.broadinstitute.org/cancer/software/GENE-E).

We also experimentally tested the responses of the *CsDof* genes to the inoculation of WMV with the cucumber line ‘Europe 8’, which is susceptible to WMV. The original WMV isolate was kindly provided by Prof. Rosario Provvidenti of Cornell University (Ithaca, New York, USA). The virus was maintained in plants of *Cucurbita pepo*. For inoculum preparation, diseased leaves were washed with ddH_2_O, and the suspension was diluted in 0.2 mol • L^−1^ phosphoric acid buffer (pH 7.0) to a concentration of 1:3 (W/V). We used the leaf inoculation method of friction. Seeds of “Europe 8” were soaked in 55 °C warm water for 4 h for disinfection. Then, the seeds were germinated in an incubator (at 28 °C) for 18 h and planted in 4 × 8 plugs in a greenhouse. When the second true leaf was fully expanded, we sprinkled a small amount of 600 ~ 800 mesh emery after frictional artificial inoculation, flushed it with clear water, and used the phosphoric acid buffer to inoculate the health leaves as a control. We set up 3 duplications, and 10 cucumber seedlings for each. Both the inoculated and control seedlings were maintained in an insect-free growth chamber with 25 ~ 30 °C and at 100% RH (Relative Humidity). Leaf samples were harvested at 0, 1, 3, 6, 9, 12, 18 and 24 days post-inoculation (dpi), which were used to extract intercellular fluid or gene expression analysis.

To investigate the *C*s*Dof* gene expression, total RNA was extracted using a Huayueyang Quick RNA isolation Kit (Cat. No.: ZH120, Huayueyang Biotechnology, Beijing, China) following the manufacturer’s procedure. To remove trace DNA contamination, DNase (Cat. No.: D2270A, TaKaRa Biotechnology, Dalian, China) was added to the total RNA samples. The qualities and quantities of RNA were determined using agarose gel electrophoresis and a NanoDrop ND-2000 Spectrophotometer (Thermo Fisher Scientific Inc., USA). For cDNA synthesis, 1 μg high-quality total RNA was reverse-transcribed with oligodT and random primers with Super Script III Reverse Transcriptase (TaKaRa, Dalian, China) according to the manufacturer’s instructions.

For qRT-PCR analysis, the specific primers for each *CsDof* gene were designed according to the *Dof* gene sequences using Primer 3 online (http://primer3.ut.ee/) (Supplementary Table S7). The cucumber ubiquitin extension protein gene (primer sequences: 5′-GGCAGTGGTGGTGAACATG-3′ and 5′-TTCTGGTGATGGTGTGAGTC-3′) was used as the reference gene^47^. qRT-PCR reactions were performed using the SYBR Premix Ex TaqTM kit (TaKaRa, Dalian, China) and a Roche LightCycler 480. All qRT-PCR experiments were performed with three biological and three technical replications. Relative gene expression was calculated using the 2^−ΔΔCt^ method. Then, the data were compiled to make a heatmap via Gene-E 3.0.240 (www.broadinstitute.org/cancer/software/GENE-E).

## Additional Information

**How to cite this article**: Wen, C.-l. *et al*. Identification and characterisation of Dof transcription factors in the cucumber genome. *Sci. Rep*. **6**, 23072; doi: 10.1038/srep23072 (2016).

## Supplementary Material

Supplementary Information

Supplementary Table S1

Supplementary Table S2

Supplementary Table S3

## Figures and Tables

**Table 1:** Information of 36 *CsDof* gene homologs in the cucumber genome.

*Dof* gene	Gene Name	Chr.	Group	pI isoelectric point	Molecular weight (Da)	Protein length (aa)	Subnuclear Localisation
*CsDof04*	*Csa1M039960.1*	1	A	8.93	16906.71	150	Nuclear
*CsDof19*	*Csa3M849890.1*	3	A	8.74	21879.22	202	Nuclear
*CsDof02*	*Csa1M015610.1*	1	A	8.16	36172.78	338	Nuclear
*CsDof32*	*Csa6M497370.1*	6	B1	8.38	34722.16	321	Nuclear
*CsDof12*	*Csa3M115040.1*	3	B1	8.61	28355.41	257	Nuclear
*CsDof31*	*Csa6M490130.1*	6	B1	8.76	35006.63	334	Nuclear
*CsDof24*	*Csa5M515000.1*	5	B1	8.64	39115.96	361	Nuclear
*CsDof15*	*Csa3M734310.1*	3	B1	9.59	36502.48	344	Nuclear
*CsDof20*	*Csa4M295460.1*	4	B1	9.51	31280.76	284	Nuclear
*CsDof23*	*Csa5M182030.1*	5	B1	9.2	34059.98	321	Nuclear
*CsDof10*	*Csa1M505950.1*	1	B1	9.42	37451.5	343	Nuclear
*CsDof27*	*Csa6M191030.1*	6	B1	9.48	38567.45	345	Nuclear
*CsDof07*	*Csa1M074970.1*	1	B2	7.34	38945.54	351	Nuclear
*CsDof35*	*Csa6M517950.1*	6	B2	9.26	27567.44	251	Nuclear
*CsDof06*	*Csa1M074960.1*	1	C1	9.32	19518.43	182	Nuclear
*CsDof05*	*Csa1M046080.1*	1	C1	8.6	29647.36	257	Nuclear
*CsDof33*	*Csa6M517150.1*	6	C1	8.97	25761.73	238	Nuclear
*CsDof08*	*Csa1M089510.1*	1	C1	7.24	34769.04	312	Nuclear
*CsDof29*	*Csa6M338090.1*	6	C1	8.41	29224.21	266	Nuclear
*CsDof13*	*Csa3M119420.1*	3	C2.1	9.08	18124.01	171	Nuclear
*CsDof22*	*Csa5M148660.1*	5	C2.1	8.43	34257.96	314	Nuclear
*CsDof14*	*Csa3M238150.1*	6	C2.1	8.6	24837.77	225	Nuclear
*CsDof26*	*Csa6M103520.1*	6	C2.1	9.03	27444.34	247	Nuclear
*CsDof21*	*Csa5M139210.1*	5	C2.1	9.03	28042.45	262	Nuclear
*CsDof16*	*Csa3M782640.1*	3	C2.2	6.64	28387.7	255	Nuclear
*CsDof28*	*Csa6M289740.1*	6	C2.2	4.8	27887.79	248	Nuclear
*CsDof09*	*Csa1M475990.1*	1	D1	8.56	20590.39	180	Nuclear
*CsDof36*	*Csa6M519570.1*	6	D1	8.89	19435.01	173	Nuclear
*CsDof01*	*Csa1M009790.1*	1	D1	5.61	51099.29	467	Nuclear
*CsDof03*	*Csa1M033250.1*	1	D1	7.16	47066.7	430	Nuclear
*CsDof17*	*Csa3M782640.1*	3	D1	5.89	54269.34	503	Nuclear
*CsDof25*	*Csa5M605720.1*	5	D1	8.67	47285.63	432	Nuclear
*CsDof18*	*Csa3M825020.1*	3	D1	5.95	55151.21	502	Nuclear
*CsDof34*	*Csa6M517940.1*	6	D2	9	29307.7	260	Nuclear
*CsDof11*	*Csa2M360720.1*	2	D2	8.21	25961.43	249	Nuclear
*CsDof30*	*Csa6M490030.1*	6	D2	8.6	24644.44	239	Extracellular
