# Supplementary material for: Identification and characterisation of Dof transcription factors in the cucumber genome
Source: Sci Rep. 2016 Mar 16;6:23072. doi: 10.1038/srep23072 (PMC4793291; doi:10.1038/srep23072)
Supplement: Supplementary Information [file srep23072-s1.pdf]

Identification and characterisation of Dof transcription factors in the cucumber genome

Chang-long Wen[1,2†*], Qing Cheng[1,2†], Liqun Zhao[3], Aijun Mao[1,2], Jingjing Yang[1,2], Shuancang Yu[1,2], Yiqun Weng[4], Yong Xu[1,2]

Supplementary Information：

Supplementary Figure S1；

Supplementary Figure S2；

Supplementary Table S2；

Supplementary Table S3；

Supplementary Table S6；

Supplementary Table S7；

Supplementary Figure S1:

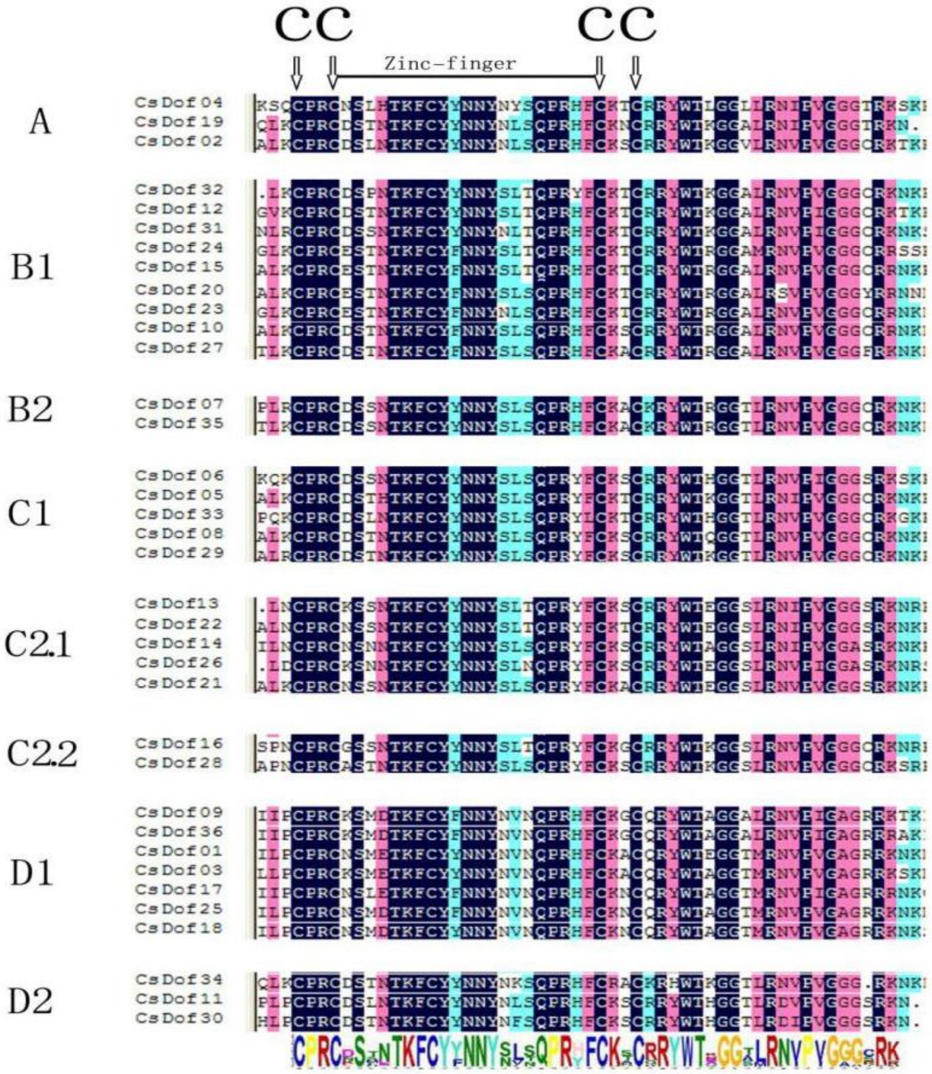

Supplementary Figure S1. Multiple sequence alignment of CsDof proteins in cucumber

Supplementary Figure S2:

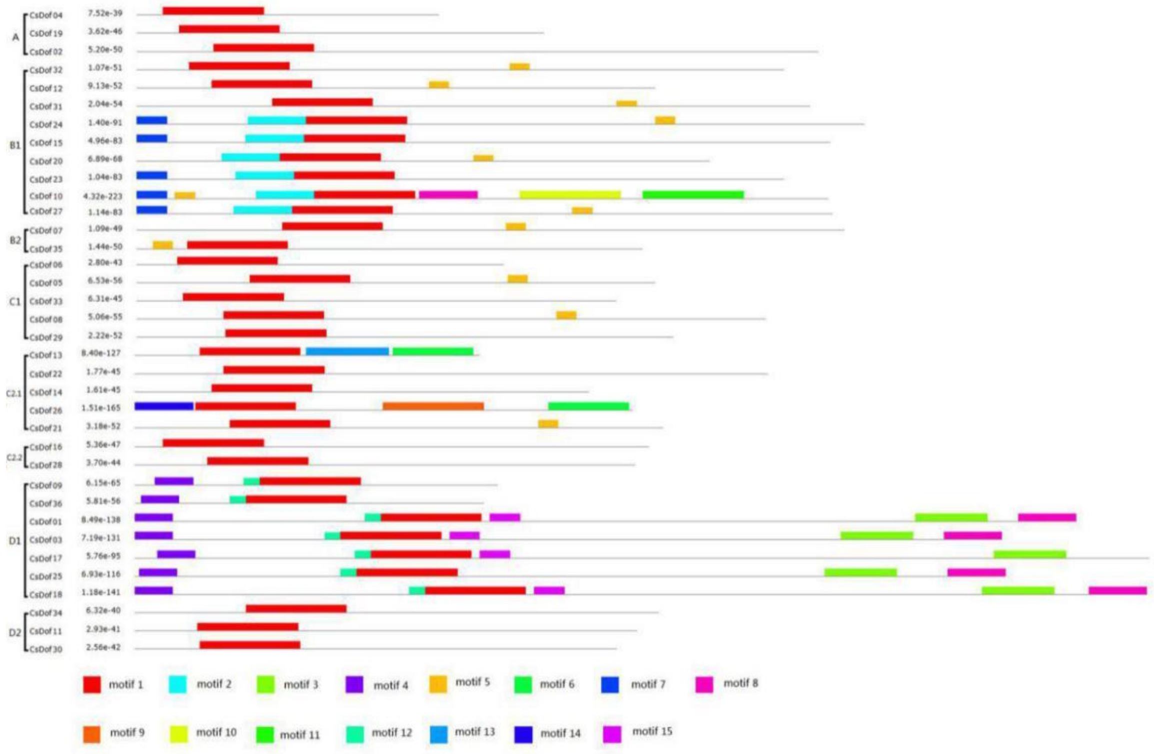

Supplementary Figure S2. Common motifs of CsDof family proteins in cucumber. Dof domains are represented by striped boxes; the red motif 1 was presenting the conserved Dof motif.

Supplementary Table S2：

The Duplication events of *CsDof* genes in cucumber genome

	Gene 1	Chr.	Gene 2	Chr.	E-value	Identities (%)	Positives (%)	Ks	Ka	Ka/Ks	Mya
Segment duplication events	*CsDof01*	1	*CsDof03*	1	e-120	50%	63%	1.4857	0.3003	0.2021	11.43
	CsDof02	1	*CsDof04*	1	2e-26	56%	72%	1.0494	0.5106	0.4865	8.072
	CsDof07	1	*CsDof35*	6	1e-40	39%	51%	1.1711	0.4812	0.4109	9.008
	CsDof09	1	*CsDof36*	6	3e-33	64%	80%	1.5161	0.3444	0.2272	11.66
	CsDof20	4	*CsDof24*	5	1e-41	46%	56%	1.2564	0.5983	0.4762	9.665
	CsDof03	1	*CsDof25*	5	5e-71	38%	53%	0.6031	0.6211	1.0298	4.639
Tandem duplication events	*CsDof06*	1	*CsDof07*	1	1e-30	40%	54%	1.8735	0.5626	0.3003	14.41
	CsDof34	6	*CsDof35*	6	2e-31	58%	73%	1.3398	0.7205	0.5378	10.31

Supplementary Table S3：

Amino acid composition of the cucumber Dof motifs.

Motif No.	Sites	E-value	Amino acid sequence composition of motif	Width (aa)	llr
Motif1	39	2.8e-1793	CPRC[DN]S[TS]NTKFCY[YF]NNY[SN]L[SNT]QPR[HY]FCK[ST]CRRYWT[KR]GG[TAS]LRN[VI]P[VI]GGG[CSR]R[KR]	50	5147
Motif2	7	1.2e-073	G[GV]GVGS[IV]R[PR]GSM[AT]DRAR[ML]A[NK][IV]P[QL]PE[AT][AG]LK	29	484
Motif3	5	9.6e-048	[KQY][EKRST][EN][GKL][CHS][VL][LGVW][ITV]P[KR]T[LM][RK][IV]D[DN]P[NSG]EA[AS][KR]S[SCP]IW[AEST]T[LF]GI[KH]	32	394
Motif4	7	1.0e-022	ML[DE]SKDP[AG]IKLFG[KR]TIPL[PQ]	19	281
Motif5	13	2.9e-022	D[LQS][HN]HHH[HQ][QP]Q[QT]	10	269
Motif6	4	4.7e-023	G[HI][GN][GS][FV][DG][CG][RY][IN][LP][LN][LQ][LM]P[FY][QS][EG][LR][EN][QT][AQ][AS][AV][AE][EP]N[ES]G[DN][HN][GQ][NY]W[NS][EG]M[FL]GGG	40	374
Motif7	6	3.3e-021	M[DV]FSS[VL]PI[YF]LDPP[NP][WN]	15	220
Motif8	2	1.2e-017	TQIPVGLRDLNLADHDHLPKVENVNYNNFAFGFRGLSSFIPNLMPNSNHI	50	290
Motif9	2	3.7e-016	VAATMAGGNESQVMNNHSNSPTTTTIPLHSSAENLIGHLQPQHPHLSFMA	50	287
Motif10	4	3.7e-015	[EM][ALST][SW][PS][VL]LQ[AV]NPAA[LF]SR[SA][IL][KTV]F[HR]E	21	210
Motif11	2	3.8e-014	ETQPGLYPFQISGGDGDENNTTNSILTPISRGTHLPPVKIEETQVLNLLK	50	284
Motif12	7	7.5e-013	K[KR]P[DE]KI[LI]P	8	150
Motif13	2	2.0e-012	PHGSVSGSGSVHHPQPQPQVYQAQDLNLGFATTAEATTAMD	41	236
Motif14	2	7.4e-012	MAEKNPNYPCKLEMKVAKATTTKAPKEQL	29	176
Motif15	4	1.2e-010	K[NQS][SL]AS[HQY][YH]R[QH]I[IT][IV]S[ES][AE][LG][QEV][AH][AT][RQT][IRT]	21	204

Supplementary Table S6:

The *AtDof* genes informations

Dof gene	Gene Name	Chomosome	Group
AtDof1.1	*At1g07640*	1	B1
AtDof1.2	*At1g21340*	1	C2.2
AtDof1.3	*At1g26790*	1	D1
AtDof1.4	*At1g28310*	1	B2
AtDof1.5	*At1g29160*	1	D1
AtDof1.6	*At1g47655*	1	D2
AtDof1.7	*At1g51700*	1	A
AtDof1.8	*At1g64620*	1	C2.1
AtDof1.10	*At1g69570*	1	D1
AtDof2.1	*At2g28510*	2	C1
AtDof2.2	*At2g28810*	2	B1
AtDof2.3	*At2g34140*	2	D1
AtDof2.4	*At2g37590*	2	B1
AtDof2.5	*At2g46590*	2	C2.1
AtDof3.1	*At3g21270*	3	A
AtDof3.2	*At3g45610*	3	C1
AtDof3.3	*At3g47500*	3	D1
AtDof3.4	*At3g50410*	3	D2
AtDof3.5	*At3g52440*	3	C2.2
AtDof3.6	*At3g55370*	3	B1
AtDof3.7	*At3g61850*	3	C2.1
AtDof4.1	*At4g00940*	4	C2.1
AtDof4.2	*At4g21030*	4	C3
AtDof4.3	*At4g21040*	4	C3
AtDof4.4	*At4g21050*	4	B2
AtDof4.5	*At4g21080*	4	C3
AtDof4.6	*At4g24060*	4	C2.1
AtDof4.7	*At4g38000*	4	B2
AtDof5.1	*At5g02460*	5	B1
AtDof5.2	*At5g39660*	5	D1
AtDof5.3	*At5g60200*	5	C1
AtDof5.4	*At5g60850*	5	A
AtDof5.5	*At5g62430*	5	D1
AtDof5.6	*At5g62940*	5	C1
AtDof5.7	*At5g65590*	5	B2
AtDof5.8	*At5g66940*	5	D2

Supplementary Table S7：

Primers for *CsDof* genes qRT-PCR analysis

Dof gene	Forward(5'-3')	Reverse(5'-3')
Actin	GGCAGTGGTGGTGAACATG	TTCTGGTGATGGTGTGAGTC
CsDof01	CCGCTAAGAGTTCGATATGGGAAAC	CCGCTAAGAGTTCGATATGGGAAAC
CsDof02	GAGGAGGAGGAGGGTATATGGATCA	CAGTATGCTTGATCAACGGCTGTAG
CsDof03	CAGTATGCTTGATCAACGGCTGTAG	CAGTATGCTTGATCAACGGCTGTAG
CsDof04	GTTGTCCAGTCCGTTGAAGTTGAAT	GTTGTCCAGTCCGTTGAAGTTGAAT
CsDof05	TCATCTTCTTCATGGCCCTTCTTCA	TCATCTTCTTCATGGCCCTTCTTCA
CsDof06	AGGACCTCTTGATCTTGCAACTTCT	TGTGTTGTTGATGGGAGTAGCAGTA
CsDof07	TGGGATGAAGAGGTTAAATTGGGAA	CATGATGCCAATTTTGAGAGGTCCA
CsDof09	ATCGTCGTTGTGGTTGTGTTTGAAT	ATCGTCGTTGTGGTTGTGTTTGAAT
CsDof10	GTTTGTACCCGTTTCAAATTAGCGG	GTTTGTACCCGTTTCAAATTAGCGG
CsDof11	TTTGACGATATGGGGTTCGCTCTAG	TTTGACGATATGGGGTTCGCTCTAG
CsDof12	TTTGACGATATGGGGTTCGCTCTAG	TTTGACGATATGGGGTTCGCTCTAG
CsDof13	ACTTGGGGTTTGCAACGACG	CCAATACCCATTATTACCGGAATTCTCTTC
CsDof14	GCTGAGAAGTAATGGCATAATGTCTCA	GGTTGAAAGTTGCTTGAAATCCTCC
CsDof15	GGTTGAAAGTTGCTTGAAATCCTCC	GGTTGAAAGTTGCTTGAAATCCTCC
CsDof17	ATGTCTGTGCATCTCAAACATCCAC	ATGTCTGTGCATCTCAAACATCCAC
CsDof18	ACGAGCAGCAGAAAGATACCAAAAC	ACGAGCAGCAGAAAGATACCAAAAC
CsDof19	ACGAGCAGCAGAAAGATACCAAAAC	ACGAGCAGCAGAAAGATACCAAAAC
CsDof20	ACGAGCAGCAGAAAGATACCAAAAC	GCTTTCCCACATTCACCTCTACTTG
CsDof21	GCTTTCCCACATTCACCTCTACTTG	CATGTCCTCCCCTATTCTCCTCATG
CsDof22	TTGGATTCTCTCTCGACGGATATGG	TTGGATTCTCTCTCGACGGATATGG
CsDof23	TTGGATTCTCTCTCGACGGATATGG	ACTCCATGCATTTACATCGTTTCCG
CsDof24	TTAGCTCATCATCACCATCCTCCTC	GCTGATGATGGGAGTGAATTAGGGA
CsDof25	AAAGCTGGTGGTGGAAGTACAAGTA	AAAGCTGGTGGTGGAAGTACAAGTA
CsDof26	AAAGCTGGTGGTGGAAGTACAAGTA	CCCAAGCATCTCACTCCAATTTTGA
CsDof27	GGTGCTAACTCATTACAACAAGCCA	AGCATTGTTCCTTGTTCTCGGTTTT
CsDof28	AGCATTGTTCCTTGTTCTCGGTTTT	TAAAATTCCAGCTCTCTCTTCCAGC
CsDof29	TAAAATTCCAGCTCTCTCTTCCAGC	CTCCCACTTGACAGAACTGATCAGA
CsDof30	GGCTATGGGACGGCTAGTTCTAG	CAAACAATCGGCAGACCCAACA
CsDof31	CAAACAATCGGCAGACCCAACA	CATTCGTTACTCCCACAGATCCCT
CsDof32	CATTCGTTACTCCCACAGATCCCT	CGGTAAATGGGTGTATGGATGCAAA
CsDof33	TTGAAACCCACAGAAACAGAGCAAC	CATGGGATGTTTAGATCAGTTGCGG
CsDof34	CATGGGATGTTTAGATCAGTTGCGG	ACAAAATCCCGGCATATAATCAGGC
CsDof35	ACAAAATCCCGGCATATAATCAGGC	ACAAAATCCCGGCATATAATCAGGC
CsDof36	ACAAAATCCCGGCATATAATCAGGC	AATCTTCCGGCGTTTGAAAGGAAAA
